# Preface – 11^th^ISTIB: Facets of terrestrial isopod biology

**DOI:** 10.3897/zookeys.1101.86064

**Published:** 2022-05-18

**Authors:** Pallieter De Smedt, Stefano Taiti, Spyros Sfenthourakis, Ivanklin Soares Campos Filho

**Affiliations:** 1 Forest & Nature Lab, Ghent University, Geraardsbergsesteenweg 267, B-9090 Melle (Gontrode), Belgium Ghent University Melle Belgium; 2 SPINICORNIS, Mispeldonk 2, B-2820 Bonheiden, Belgium SPINICORNIS Bonheiden Belgium; 3 Istituto di Ricerca sugli Ecosistemi Terrestri, Consiglio Nazionale delle Ricerche, Via Madonna del Piano 10, 50019 Sesto Fiorentino (Florence), Italy Istituto di Ricerca sugli Ecosistemi Terrestri, Consiglio Nazionale delle Ricerche Florence Italy; 4 Museo di Storia Naturale dell’Università di Firenze, Sezione di Zoologia “La Specola”, Via Romana 17, 50125 Florence, Italy Università di Firenze Florence Italy; 5 Department of Biological Sciences, University of Cyprus, University Campus, 1 Panepistimiou Ave., 2109 Aglantzia, Nicosia, Cyprus University of Cyprus Nicosia Cyprus

The 11^th^ International Symposium on Terrestrial Isopod Biology (ISTIB) was intended to take place in 2020 but a world health crisis decided differently. Towards the end of 2019, cases of pneumonia caused by a new coronavirus (SARS-CoV-2) were reported from Wuhan, China. The virus spread quickly and was declared a pandemic in March 2020. At that time, nobody could foresee the severe consequences this pandemic caused in our daily lives. Many countries went into different forms of lockdown and international travel became impossible. Therefore, it was justly decided to postpone the 11^th^ISTIB to 2021. However, countries around the world went from a first wave, to a second and a third one, and it became clear that an in-person ISTIB would not be possible in 2021 either. The last ISTIB (10^th^) was held in 2017 in Budapest, Hungary (Hornung et al. 2018), thus repeated postponement of the next meeting meant a gap of at least five years for the terrestrial isopod community to meet again in person. As the world was still suffering from subsequent waves of SARS-COV-2 virus, the terrestrial isopod community decided to meet virtually in 2021 to share their current research and stimulate new collaborations.

*Spinicornis*, the Belgian Terrestrial Isopod Group, took on the job of hosting this online edition at Ghent University, Ghent, Belgium. *Spinicornis* was established in 2014 with the mission of continuously collecting distribution data of terrestrial isopods from Belgium (https://www.spinicornis.be). After five years, they made inventories in every 10×10 km^2^ of the Belgian territory, catching up with neighbouring countries on the knowledge of terrestrial isopod distribution. The data were recently published in an ecological atlas on the terrestrial isopods of Belgium (De Smedt et al. 2020) and in this special issue. Having reached their main goal, this terrestrial isopod group could put all their energy in the organisation of the first online edition of ISTIB. The editors of this special issue thank the members of *Spinicornis*, and in particular Stijn Segers, Gert Arijs, and Pepijn Boeraeve, for their instrumental contribution on practical issues of the congress. *Spinicornis* co-organised the symposium with the Forest & Nature Lab (ForNaLab), Department of Environment of the Faculty of Bioscience Engineering from Ghent University (https://www.ugent.be/bw/environment/en/research/fornalab). ForNaLab studies interactions of ecological processes in terrestrial ecosystems focusing on forests and grasslands in temperate regions. The research is tightly linked to forest and nature management and policy. During the past decade, the lab increasingly incorporated invertebrates, including terrestrial isopods, in their research as important components of ecosystem functioning.

**Figure 1.**
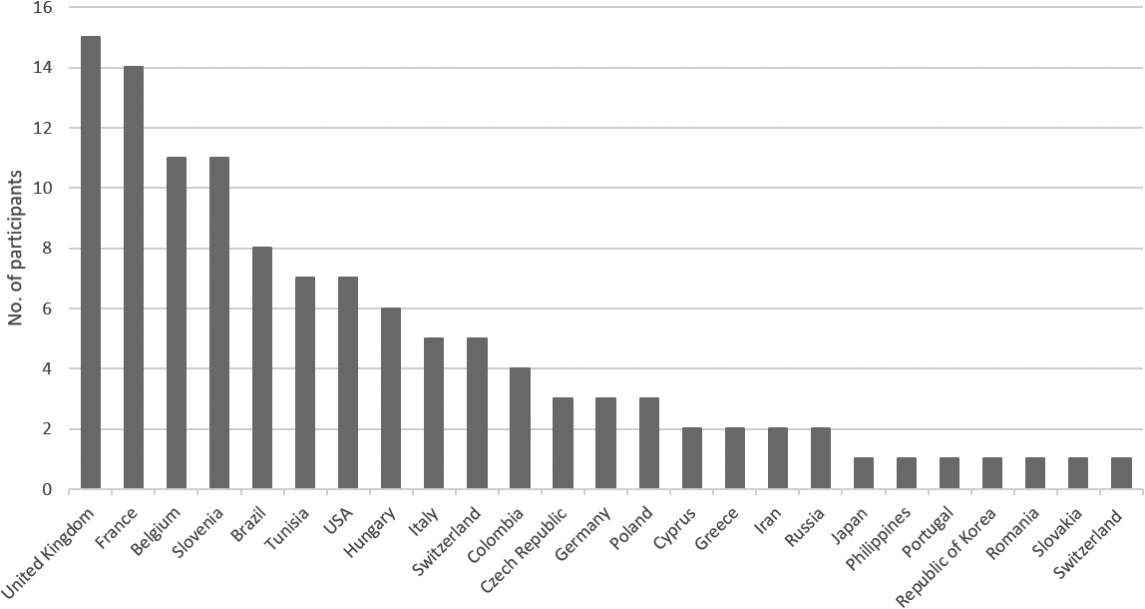
Number of participants per country.

**Figure 2.**
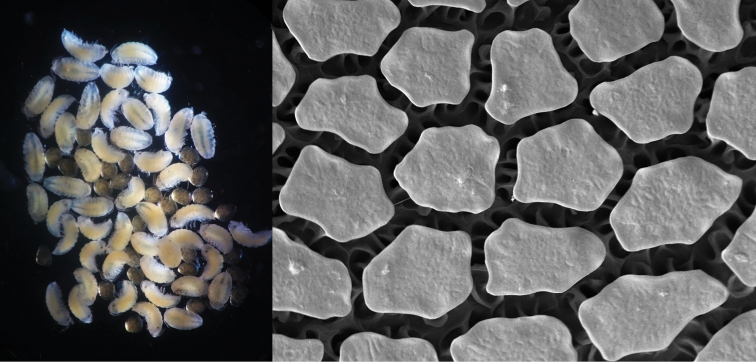
Two of the award-winning photographs of the ISTIB photo competition across the jury and public voting **A** offspring of *Armadillidiumvulgare* (Latreille, 1804), winner in the category “Terrestrial isopods”; photograph by Cybèle Prigot **B** the perispiracular cuticle in *Porcelliolaevis* Latreille, 1804, winner in the category “Lab research”; photograph by Miloš Vittori.

The virtual meeting event was a success clearly demonstrating the need of the terrestrial isopod community to stay in contact and seek new collaboration within and across countries. A total of 117 participants from 25 countries worldwide attended the symposium (Fig. 1). The participants listened to 29 oral presentations, including six invited keynote talks by leading experts on different aspects of terrestrial isopod biology. Additionally, 23 posters were presented and discussed in virtual poster rooms. The oral and poster presentations were placed in one of the following six sessions: 1) Taxonomy, phylogeny, and faunistics; 2) Distribution and biogeography; 3) Anatomy and physiology; 4) Host-microbial interactions; 5) Behaviour; 6) Ecology. Although this was a virtual ISTIB, a social event was organized, where participants could meet in rooms and chat in smaller groups while enjoying their favourite drink from their home or work space. This virtual event was preceded by a photograph contest in which all participants could vote (Fig. 2).

This special issue “Facets of Terrestrial Isopod Biology” is a collection of twelve papers presented at the 11^th^ISTIB symposium covering a wide range of topics on recent terrestrial isopod research. The first research paper is a bibliometric analysis of the terrestrial isopod research from the past 70 years which shows how the bulk of terrestrial isopod research is performed and published in developed countries and concentrated in few research labs. Moreover, many of the published research papers are not indexed in “Web of Science”. This review also provides evidence that conference proceedings, such as this issue, are significant contributions to the field. The second research paper describes two species of terrestrial isopods new to science, from Brazilian caves, contributing to the knowledge of cavernicolous isopods from the Neotropical region. A presentation of the database of *Spinicornis* covers the detailed distribution of terrestrial isopods in the Belgian territory. There are three papers on terrestrial isopod behaviour: one studying reactions of terrestrial isopods to high temperature and substrate vibrations, one that studies their behaviour towards toxicity tests, and a third one is a review of the different strategies evolved in terrestrial isopods to avoid predation. An interesting and rarely studied topic is covered in a paper discussing consequences of immune priming on life history traits of *Armadillidiumvulgare* (Latreille, 1804) in the framework of host-microbial interactions. There are two morphological papers, one on the remodelling of septate junctions in the epidermis of *Porcellioscaber* Latreille, 1804 during development, and one on the first observation of intersexuality in the same species. The issue closes with two ecological papers based on nocturnal fieldwork. One paper studies the distribution of terrestrial isopods on a wall in the Czech Republic and the other studies the daily and seasonal activity patterns of the poorly studied *Porcellioalbinus* Budde-Lund, 1885 in an arid region in Tunisia. The diversity of these different papers nicely covers different facets of terrestrial isopod biology in a constantly diversifying field (Schmalfuss 2018). This special issue pays tribute to Dr Jonathan C. Wright, a dedicated scientist and terrestrial isopod enthusiast who passed away too soon. The terrestrial isopod community is grateful for his contribution to understanding the relationships between functions of terrestrial isopods and their environment.

We acknowledge that the online format has a different flow and feeling than the in-person meetings we are all accustomed to. At the same time, it provided both the organizers and participants novel symposium experiences that will probably become more common in the future. The virtual meeting also gave the opportunity for researchers from remote countries and/or with limited resources to attend. Until now, all ten previous ISTIB editions were held in or nearby Europe. We keep our hopes high to meet in person again or at least in a hybrid format at the 12^th^ISTIB, to further advance the research on these intriguing invertebrates that we all highly value as important components of the functioning of our natural world.
